# Providing care under extreme adversity: The impact of the Yemen conflict on the personal and professional lives of health workers

**DOI:** 10.1016/j.socscimed.2021.113751

**Published:** 2021-03

**Authors:** Shatha Elnakib, Sarah Elaraby, Fouad Othman, Huda BaSaleem, Nagiba A. Abdulghani AlShawafi, Iman Ahmed Saleh Al-Gawfi, Fouzia Shafique, Eman Al-Kubati, Nuzhat Rafique, Hannah Tappis

**Affiliations:** a Johns Hopkins Bloomberg School of Public Health, Baltimore, MD, USA; b Taiz University, Taiz, Yemen; c Aden University, Aden, Yemen; d Sana'a University, Sana'a, Yemen; e UNICEF HQ, New York, USA; f UNICEF Yemen Country Office, Sana'a, Yemen; g Alexandria University, Alexandria, Egypt

**Keywords:** Yemen, War, Conflict, Violence, Frontline health worker, Humanitarian, Health service delivery

## Abstract

The war in Yemen, described as the world's ‘worst humanitarian crisis,’ has seen numerous attacks against health care. While global attention to attacks on health workers has increased significantly over the past decade, gaps in research on the lived experiences of frontline staff persist. This study draws on perspectives of frontline health workers in Yemen to understand the impact of the ongoing conflict on their personal and professional lives. Forty-three facility-based health worker interviews, and 6 focus group discussions with community-based health workers and midwives were conducted in Sana'a, Aden and Taiz governorates at the peak of the Yemen conflict. Data were analysed using content analysis methods. Findings highlight the extent and range of violence confronting health workers in Yemen as well as the coping strategies they use to attenuate the impact of acute and chronic stressors resulting from conflict. We find that the complex security situation – characterized by multiple parties to the conflict, politicization of humanitarian aid and constraints in humanitarian access – was coupled with everyday stressors that prevented health workers from carrying out their work. Participants reported sporadic attacks by armed civilians, tensions with patients, and harassment at checkpoints. Working conditions were dire, and participants reported chronic suspension of salaries as well as serious shortages of essential supplies and medicines. Themes specific to coping centered around fatalism and religious motivation, resourcefulness and innovation, and sense of duty and patriotism. Our findings demonstrate that health workers experience substantial stress and face various pressures while delivering lifesaving services in Yemen. While they exhibit considerable resilience and coping, they have needs that remain largely unaddressed. Accordingly, the humanitarian community should direct more attention to responding to the mental health and psychosocial needs of health workers, while actively working to ameliorate the conditions in which they work.

## Highlights

• Yemen's healthworkers bear double burden, providing care amid violence and adversity.
• Healthworkers report disempowerment, distress, fear, anxiety, and burnout.
• Health workers use problem-focused and meaning-focused coping strategies.
• Healthworkers serving in conflict-affected areas need increased attention and support.

## 1 Introduction

Attacks on health care – both deliberate and indiscriminate – are a recurring feature of several contemporary conflicts, despite protections afforded to health care workers and facilities under international humanitarian law (Briody et al., 2018). Conflict has devastating effects on health systems, both directly through attacks on health infrastructure, personnel and patients, and indirectly through interferences that disrupt health care delivery (Footer et al., 2014).

Health workers, who are at the center of health systems, often unduly bear the brunt of this violence. One study demonstrated that out of 921 violent incidents affecting health care in 22 conflict-affected countries, 91% were of violence inflicted on local health workers (ICRC, 2013). Recent studies exploring the experiences of health workers in conflict and post-conflict settings have highlighted the heightened vulnerability of health workers to physical attacks, arrests, and intimidation as well as their exposure to stresses stemming from increased workload, economic hardship and poor working conditions (Footer et al., 2014, 2018; Fouad et al., 2017; Witter et al., 2017).

In Yemen, the ongoing war which has been described as the world's ‘worst humanitarian crisis’ has seen numerous attacks against health care. According to the World Health Organization (WHO), more than 100 attacks have been documented to date, ranging from repeated airstrikes and shelling of health facilities, to attacks on medical transports and supplies, as well as threats and attacks on health workers and patients (WHO, 2017).

Yemen is divided into multiple areas of territorial and political control. There are numerous parties to the conflict with diverse political and ideological foundations, sources of material support, and regional alliances. The conflict, which entered its sixth year in March 2020, is generally described as a civil war between ‘pro-government’ forces (referring to forces allied with the internationally recognized government [IRG] in the South, whose capital is Aden and which is backed by a Saudi-Led Coalition [SLC] that includes the US and UK) and ‘pro-Houthi’ forces in the North and whose capital is Sana'a. Although these are the principal belligerents, there are other parties to the complex conflict – including a Southern Resistance Movement and ISIS, among others. The Houthi movement, which long predates the ongoing civil war, began in 2004 and was joined in 2014 by forces loyal to former president Ali Abdallah Saleh (ACAPS, 2018). This alliance prompted Saudi Arabia and other members of the coalition to initiate an air campaign followed by a land campaign aimed at regaining territories controlled by Houthi forces – including the capital city of Sana'a. In 2015, the SLC took control of Aden and expanded to much of Southern Yemen, and in April of the same year, a confrontation between the SLC and Houthi forces resulted in multiple air-strikes and ground clashes in Aden and Sanaa in addition to a siege of Taiz city, (ACAPS, 2018). As a result of the siege, residents of Taiz have had restricted access to vital medical supplies, food, and water (MSF, 2017).

**Fig. 1:**
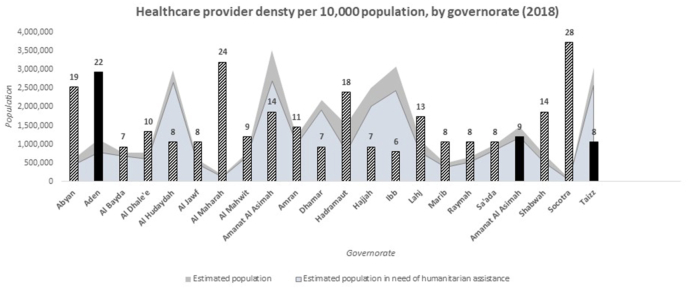
Availability of Health Workers. Data sources: Yemen Humanitarian Needs Overview 2019 (UNOCHA, 2019) and HeRAMS 2018 (WHO, 2018).

In addition to exacting a great toll on the Yemeni people, with more than 70,000 civilian casualties documented by health facilities in the period between March 2015 to October 2018 (UNOCHA, 2019), the conflict has had wide ranging implications for health service delivery and the health system at large. Impacts have included an unprecedented cholera epidemic – the largest in history – coupled with other disease outbreaks including diphtheria and dengue fever (Camacho et al., 2018). In 2019, only 51% of health facilities were fully functioning, and 35% were only partially functioning (UNOCHA, 2019). Additionally, only 10 health workers were reportedly available per 10,000 people in Yemen and shortages in specialists were particularly pronounced (Fig. 1). The loss of skilled health workers has been exacerbated by the suspension of salaries of approximately 1.5 million state employees including health workers. Blockades on ports of entry have obstructed the passage of important supplies, causing a chronic shortage of commodities and equipment (Coppi, 2013). Against this backdrop, health workers have had to provide services while they themselves, and the facilities in which they work, are subjected to violence(see .

Despite increasing global attention to attacks on health care and a burgeoning body of literature exploring the personal and professional challenges faced by health workers in conflict settings, studies continue to focus predominantly on the impact of conflict on the health system as a whole and its resiliency in the face of adversity rather than on the lived experiences of frontline health workers, and the coping mechanisms they employ. To supplement existing knowledge about the plight of health workers serving in conflict-affected settings, this study draws on perspectives of health care workers in Yemen to understand the impact of the ongoing conflict on aspects of their personal and professional lives.

We pull on theories of stress and resilient coping, which support the idea that some individuals are able to maintain stable ‘healthy levels of psychological and physical functioning’ when faced with highly disruptive events (Bonanno, 2004). In the context of the health workforce, resilient health providers are able to manage their own stress constructively, employing various coping mechanisms that are instrumental to the overall resilience of the health system (Witter et al., 2017). Lazarus and Folkman's theory of stress and coping classifies coping strategies into problem-focused, emotion-focused and, more recently, meaning-focused coping (Folkman, 2008; Lazarus, 1984). They define problem-focused coping as coping that is oriented with managing or tackling a problem that is perceived as fixable or amenable to change (Lazarus, 1984). Emotion-focused coping signifies coping in which individuals regulate their emotional response to problems, particularly when they have assessed that little can be done to alter challenging conditions. Meaning-focused coping refers to coping in which individuals draw on their internalized beliefs and value systems to stimulate and sustain well-being during extreme adversity (Folkman, 2008).

We additionally examine another facet of coping within the context of traumatic experiences drawing from the concept of self-efficacy. In social cognitive theory, self-efficacy refers to an individual's perception or judgment of their own ability to perform a certain activity in order to reach a certain outcome (Bandura et al., 1999). Benight and Bandura re-introduced the concept of coping self-efficacy in the face of adversity and defined it as “the perceived capability to manage one's personal functioning and the myriad environmental demands of the aftermath occasioned by a traumatic event”.

Using foundational concepts such as coping and self-efficacy as theoretical points point of departure, we examine how the conflict in Yemen impacted the lived experience of health workers and their coping capacities.

## 2 Methods

### 2.1 Study design

This is a qualitative study, drawing on primary data collection conducted as part of a case study examining how reproductive, maternal, newborn, child and adolescent health and nutrition (RMNCAH + N) services have been delivered in Yemen since the start of the crisis in 2015 (Tappis, 2020). The study, guided by a common protocol for case studies conducted by the BRANCH (Bridging Research & Action in Conflict Settings for the Health of Women and Children) Consortium in multiple conflict-affected countries, included a review of documents and datasets reporting RMNCAH + N service coverage and implementation strategies since 2000 and primary data collection in three governorates of Yemen in late 2018 (Ataullahjan, 2020). Broader case study findings are published elsewhere (Tappis, 2020 #2).

Although the original case study was not designed with the intention to investigate the impacts of the conflict on health workers’ lived experiences, our interviews included questions on how the conflict affected participants and their families and, on many occasions, participants volunteered compelling accounts of their experiences providing care amidst extreme adversity. This paper thus purposefully examines the impacts of the ongoing conflict on health workers by analyzing semi-structured in-depth interviews (IDIs) conducted with facility-based providers and focus group discussions (FGDs) conducted with community-based providers.

### 2.2 Study setting

We conducted IDIs and FGDs in three of Yemen's 22 governorates: Sana'a City *(Amanat al Asimah)*, Aden and Taiz. Study sites were selected in consultation with the Ministry of Public Health and Population and UNICEF, considering the political authority, nature of the conflict, population size, level of humanitarian presence, and accessibility for data collection.

Sana'a city *(Population:* 3 million with 2 million in need of humanitarian assistance in 2018) is an urban governorate. It is the capital of Yemen recognized in the Constitution, and its largest city. Sana'a City has been under Houthi-led government control since September 2014.

Aden (*Population:* 925,000 with 800,000 in need of humanitarian assistance in 2018) is an urban governorate and the second largest city in Yemen. Aden was under Houthi control from March–July 2015. Since then, the city has been under the control of Southern Resistance elements affiliated with the internationally recognized government led by President Hadi and backed by the Saudi-led Coalition.

Taiz governorate is Yemen's most populous governorate (*Population:* 3.2 million with 2.9 million in need of humanitarian assistance in 2018), home to Yemen's third largest city (Taiz city), as well as rural highland and lowland areas. In 2013, approximately 80% of the governorate population lived in rural areas and 20% in urban. Taiz has been a frontline of the conflict since March 2015, with multiple changes in control of territory within the governorate. At the time of data collection, 13 districts were controlled by the internationally recognized government, 6 districts controlled by the Houthi-led government, and 4 districts actively contested.

### 2.3 Data collection

Primary qualitative data collection for the broader case study included semi-structured IDIs and FGDs with government officials, representatives of humanitarian organizations and health workers purposively selected to capture a diverse range of perspectives on RMNCAH + N services in the three governorates.

The data collection guides were developed by US-based co-investigators based on the BRANCH Consortium's global case study protocol and objectives. US-based co-investigators sought feedback and input from Yemeni co-investigators both remotely and during a 3-day workshop in Cairo, Egypt prior to data collection. All tools were translated and back-translated to Arabic before training of data collectors.

For the purpose of this paper, we relied mainly on findings from 43 facility-based healthcare providers' interviews, and the 6 FGDs with community-based health workers and midwives that described personal accounts of their experience delivering care in this context (Table 1). We used findings from the desk review and other interviews with health government officials and humanitarian agency staff only to expand contextual understanding and supplement accounts regarding providers’ experiences and exposures. All healthcare providers interviewed in this study were Yemeni. With the exception of a few expatriates, humanitarian staff were also Yemeni nationals. We sought to include the perspectives of a wide range of health workers and so included 2 reproductive healthcare providers, 16 maternal and newborn healthcare providers, 3 immunization specialists, 5 child healthcare providers, 5 nutrition specialists and 12 healthcare managers or generalists. Facility-based providers were recruited from public health facilities and interviews occurred mainly at their place of work.

**Table 1:** Study participants.

Type of provider	Sana'a City	Aden	Taiz	TOTAL
Facility-based	11 IDIs [3 facilities] − 5 hospital staff, 6 primary healthcare facility staff	13 IDIs [3 facilities] − 6 hospital staff, 7 primary healthcare facility staff	19 IDIs [5 facilities – including 3 in IRG-controlled areas and 2 in Houthi-controlled areas] − 13 hospital staff, 6 primary healthcare facility staff	43 IDIs [11 facilities]
Community-based	2 FGDs (19 participants) − 10 community midwives − 9 community health volunteers	2 FGDs (20 participants) − 10 community midwives − 10 community health volunteers	2 FGDs (22 participants) − 10 in IRG-controlled area − 12 in Houthi-controlled area	6 FGDs (61 individuals trained as community midwives or community health volunteers)
Total	30 individuals	33 individuals	41 individuals	104 individuals

Interviews and FGDs were conducted by Yemeni study team members and 14 trained local research assistants (4 in Aden, 4 in Sana'a, 6 in Taiz) . The training material was developed by US-based study team members in conjunction with Yemeni co-authors who carried out the training in-country. The five-day training covered study objectives, qualitative research principles, case study design, interviewing techniques, reflexivity, notetaking and reporting, use of recording devices, as well as research ethics, and a brief technical orientation. The training was also used to test and adapt the study guides. Although the training was standardized, the research assistants hired varied in terms of their health background, experience in qualitative research, as well as communication and writing skills, which reflected on the varying quality of some interviews.

All data collection activities occurred between September–October 2018. Most participants did not know the research assistants prior to data collection; however, many were very familiar with the study team leads for each governorate who are respected and well-known researchers in the RMNCAH + N field in Yemen.

All interviews and FGDs were conducted in Yemeni dialect Arabic and were audio recorded. In addition, data collectors submitted field notes with information regarding the description of the interview setting, participants, contextual information as well as reflexive notes. The audiorecordings were then translated and transcribed into English by a private US-based company independent of the research team. All web-based data transfers to the company were encrypted and data was stored on secure encrypted servers. The US-based transcription firm's systems controls and internal policies, including non-disclosure agreements, ensure that transcribers adhered to strict measures for human subjects research protection and client confidentiality.

During data collection, there were frequent debriefing sessions carried out in-country by the research team leads and research assistants to understand and address difficulties in questions, approaches to sampling and recruitment, as well as field challenges. In addition, US-based study team members had near-weekly conference calls with the in-country team leads to follow up study progress, changes to data collection plans, understand emerging themes, and update the guides if needed. Field notes and debriefing were also used to guide the analysis and development of the codebook.

### 2.4 Data analysis

All transcripts were compiled and analysed using qualitative content analysis methods (Moretti et al., 2011; Saldaña, 2015). The initial phase of codebook development was deductive, based on the overall case study objectives. This was followed by inductive codes added based on field notes, debriefs, and open coding of a few initial transcripts. This was an iterative process, with revisions to codes and categories as more data was analysed. We then carried out a second analysis process with a focus on the healthcare providers' experiences during the crisis. This analysis included recoding and memo-ing based on the emerging themes that were the impetus for this paper and extensive discussions with the study team, as well as a review of the literature regarding the impact of conflict on humanitarian workers and healthcare providers. All coding of transcripts occurred in NVivo 12 software. Coding was done by the first and second authors who have graduate-level training in public health and Arabic fluency.

### 2.5 Ethics

The Johns Hopkins Bloomberg School of Public Health determined that this study was not human subjects research and therefore did not require institutional review board oversight (IRB 8665). In Yemen, the study protocol was reviewed and approved by the Research and Ethics Committee at the University of Aden School of Medicine and Health Sciences] (REC-33-2018). Oral consent was obtained from all study participants before initiating data collection.

Beyond formal approvals, conducting research in a humanitarian setting is wrought with ethical challenges which require careful considerations. Guided by the Research for Health in Humanitarian Crises (R2HC) ethical framework based on a desk review of humanitarian stakeholders in 2014 (Curry et al., 2014), as well as qualitative research ethics literature (Guillemin and Gillam, 2004; Wong, 1998), the protection of both study team members and participants, and implications of research activities were considered at all stages of study design and implementation. First, we formed a study team comprised of local experts and collaborators with decades of experience in Yemen in the fields of research, healthcare, and multilateral and community-based organizations. Given the limitations on time and resources of healthcare workers in Yemen, continuous consultation with the local team took place to ensure the relevance of study to community priorities, in addition to observing the necessary respect for the cultural context and norms. Our protocol and interview guides clearly articulated benefits, harms and risks to participants. In addition, due to the different factions involved in the Yemeni conflict and the governing of the healthcare system, we worked closely with partners to ensure that the study tools and objectives avoided political sensitivities, which could endanger our team or participants, and that all language used remained as neutral as possible.

Second, during data collection, we anticipated the possibility of discussing sensitive topics that might elicit anxieties, distress or trauma memories among participants (Wong, 1998). This was limited by making sure the consent forms clearly explained the aim of the study, and that data collectors avoided asking personal questions. Participants were provided with privacy during the interviews and assured of the confidentiality of their accounts. They also were informed of their abilities to opt-out of any question and to terminate the interview at any point. The study team had no direct affiliation with the employers of participants at the time of the study to avoid power differentials that could impact rapport. Care was taken in maintaining anonymity of participants and de-identifying all interview documents. The representation of quotes was limited to the general job description and governorate, and we did not collect any personal identifiers such as gender or age. Throughout the analysis process, we continuously consulted with local partners and research team members on “meaning” and representation of findings.

## 3 Results

We begin by providing an overview of the impacts of the Yemen conflict on health workers. The interviews and FGDs revealed a myriad of impacts on providers' personal safety, mental wellbeing, and perceptions of self-efficacy. For simplicity, we organize impacts into personal and professional (Witter et al., 2017). While not perfectly demarcated, this distinction serves to highlight the ways in which the conflict has permeated different dimensions of the study participants' lives. Personal impacts encompass effects of the conflict on providers' sense of wellbeing and safety while professional impacts denote direct effects of the conflict on service provision and working conditions which in turn profoundly impact health workers’ experiences and coping strategies. We then outline coping measures employed by participants in reaction to conflict-related hardships.

## 4 Personal impact

### 4.1 Insecurity at the workplace and death in the line of duty

A general climate of insecurity pervaded the three governorates, but accounts of violence were most intense in Taiz, one of the frontlines of the conflict. There were several accounts of attacks, air strikes and various forms of violence resulting from direct state-led militant actions (either by IRG and SLC, or Houthi-related militias) which targeted health facilities. In addition, direct looting, shelling, and the haphazard destruction of hospital infrastructure by thugs and armed groups were frequently reported. This general insecurity varied across time, with many participants recounting ebbs and flows of violence as the conflict progressed.

Providers spoke to the receding role of the state due to the conflict and the proliferation of militant groups and armed civilians which led to a state of lawlessness and general insecurity. Health workers were left vulnerable to attacks at the workplace and were occasionally deliberately targeted. The perpetrators of the attacks were not always identifiable, and health workers expressed confusion at not knowing who their attackers were due to the multiplicity of militant groups that were formed during the conflict, each with distinct leadership and political affiliation. This chaos left health workers feeling unprotected at the workplace and vulnerable. One participant vividly described an attack by militants who stormed the hospital with machine guns, randomly firing bullets:

*“There were children in the nursery when the shooting happened. I tried to protect the children with my body; I embraced them and tried to hide them, to protect them from the bullets. The doctor who was working with me had a meltdown and left the job and did not come back. The situation was very tragic here in this hospital, and you can see the traces of bullets everywhere in the hospital and even in the operating room.”* -Healthcare provider, Taiz

Another participant recounted an incident in which an X-ray technician had both legs amputated during a violent confrontation.

*“A sad story is that of our x-ray technician whose legs were amputated. The situation was miserable. We had faced problems with armed militias and the role of the state had been absent. We were not protected.” -*Hospital manager/pharmacist, Taiz

Even in Aden, where conditions were relatively more stable after the reclamation of control by the IRG, participants still faced security threats by different militias and an increasingly armed populous.

Across governorates, participants were anguished by the news of their coworkers dying on the job. In several cases, these deaths were due to targeting of healthcare facilities; in others, they occurred in the community while health workers were making home visits or undertaking outreach activities. Describing the deaths of several midwives, one participant noted:

*“Two months ago, a midwife died while doing her job in the community. Six midwives have died since 2015. Midwives are exposed to indescribable danger. They are called upon to work in insecure conditions during the night and they move from one place to another. …. A midwife from Aden was called to deliver a baby in Taiz. The roads were insecure, and snipers were everywhere. While delivering the baby, they were bombed by a shell. The mother and the midwife died but the baby girl did not. She came out alive.”* - NGO program manager, Sana'a

### 4.2 Raids and shelling of homes

Providers, like many other Yemeni civilians, experienced direct violence that impacted their homes, towns, and cities. Many participants shared accounts of raids, shelling, and gunfire at or near their family residences. Several described having to flee their homes temporarily or permanently due to security concerns. As one provider in Taiz who faced personal threats by armed groups recounted:

*“A mortar shell destroyed the barrels, the pipes, and a part of the walls of my home. Whenever I hear an explosion, I feel it in my home. The explosions are so close. One of my kids used to get up terrified when the area is shelled. Nevertheless, the situation is better now than before.”* Hospital manager, Taiz

### 4.3 Disempowerment, distress, and demoralization

The relentless attacks on providers' homes and places of work coupled with the deterioration of living conditions due to the conflict profoundly impacted service providers’ sense of safety and wellbeing. Although our study did not directly assess the impact of the conflict on the mental health of providers, most participants shared an understanding that the conditions in which they were living and working had a serious impact on their mental wellbeing. Providers repeatedly mentioned overwhelming feelings of fear, anxiety, fatigue, and insecurity and described the pain of reliving violent experiences:

*“I become so nervous. When an area is bombarded while I'm at my work, I directly think of my children. I imagine that they may be dead because of the shelling. However, I am trying to overcome such thoughts. I am trying to be stronger. “*Community midwife, Sana'a

These feelings in turn impacted providers' sense of self-efficacy. There was a general feeling of helplessness and inadequacy among providers who doubted their ability to continue working under the same stressful conditions as well as their capacity to meet the needs of their patients. Less explicit but certainly present in provider accounts were feelings of shame at their own perceived incapacity. As a physician in Sana'a describes:

*“I feel inadequate in my work because sometimes I cannot provide services out of fear and anxiety. I am afraid for my personal safety and concerned about the safety of patients.”* Nurse, Sana'a

Health workers’ sense of distress was compounded by personal experiences of being unable to help their own families when they needed medical attention. Many participants described personal stories in which they could not take family members to facilities to receive care in time because of the surrounding violence or the reality of living under siege. As one healthcare provider described in Taiz:

*“I was psychologically and financially affected, and I lost one of my family members when he suffocated. We couldn't help him or go to any medical center because of the clashes, shelling and shooting, and because the area we lived in was besieged. He was in need of oxygen. His skin turned blue and he died because we couldn't move him to the hospital. All this because of the war.”* Emergency doctor and obstetrician/gynecologist, Taiz

## 5 Professional impact

### 5.1 Understaffing and turnover during insecurity and economic downturn

A recurrent theme articulated by participants was the shortage and high turnover of health workers, and the relentless pressure that left on those who continued to show up to work. Because of the conflict, many health workers were injured or killed. Many fled from conflict-affected areas to safer locations inside the country or migrated voluntarily to neighboring countries. Others were displaced to other governorates or cities. This left health facilities with few providers. Participants described the surge in demand caused by disease outbreaks like cholera and dengue, population movement, and the shutdown of several health facilities. Against this backdrop, the de-facto government suspended salaries for civil servants including health workers. Some participants reported that their salaries were interrupted up to a year at a time. In other facilities, they reported receiving a nominal payment every 6 months instead of their monthly salary. Interrupted or altogether absent remuneration coupled with hiring freezes exacerbated the health worker shortage and intensified providers’ frustrations and vulnerability. Health workers recurrently reported extreme exhaustion from being overworked and under-resourced; at times there were only one or two health workers running an entire facility. Some reported working 24 h a day because of the scarcity of health workers in their facilities.

*“We are in need of full staff including specialists, consultants, general practitioners, nurses, and midwives. We are suffering from a great shortage. I mean it is unbelievable that one midwife has to handle 40–50 patients, I need at least 4 additional midwives. We have two midwives, who each work 24 hours a day.”* Obstetrician / gynecologist, Taiz

### 5.2 Shortage of supplies and impact on patient-provider relations

The conflict led to a progressive worsening of working conditions and supply shortages. The siege in Taiz prohibited the entry of essential medical supplies and the Saudi-led coalition's naval and air restrictions further strangulated the entry-point for critical goods and medicines. Providers reported struggling to provide care in the absence of basic supplies such as oxygen, gloves, gauze, cotton, stethoscopes, cannulas, anesthesia, and antibiotics. Due to lack of resources and disruption of services, participants described feeling frustrated and powerless to provide the care they knew they were capable of if conditions were not as dire.

*“One mother came with her daughter who had a fever with a temperature higher than 39 degrees Celsius. We gave her Paracetamol and Dextrose and did everything we could but the lab was closed and we could not do anything more. Imagine that a girl dies at the age of 18 in front of you because of Malaria.”* - General practitioner/reproductive health, Aden

The absence of important supplies not only interfered with health workers’ ability to do their jobs and their sense of self-efficacy, but also cultivated tensions with community members who ascribed blame for the shortages in medicines and the deterioration in quality to service providers, accusing them in some cases of stealing the medication or hiding supplies. This erosion of trust between community members and health workers resulted in clashes and disagreements. Many providers reported that arguments with patients and tensions were commonplace.

*“Some patients insult and accuse us of stealing medicines. For instance, heart disease drugs cost 20,000–30,000 Riyals monthly, which is really expensive. When you tell the patients there are no more medicines left, directly he will ask: where did you take them? You stole them all.” -*Hospital director and general surgery specialist, Aden

On several occasions, providers reported confrontations with women who upon learning that they were pregnant, blamed health workers for the extended stockouts in contraceptives.

### 5.3 Deteriorating infrastructure

Even when health facilities were not completely destroyed, participants described the rampant destruction of infrastructure which had a profound impact on service delivery. Long and recurrent electricity cuts were commonplace. Participants reported their frustration at working with unreliable and obsolete equipment, which they said they could not trust to yield accurate results. Many described themselves as unable to provide good quality care due to the inadequacy of equipment and supplies.

*“The existing hospital equipment is ancient, and most of it has stopped working. We can't even rely on their results. They're dysfunctional.”* General practitioner, Taiz

*“Regarding the services of the hospital, they are barely working. As doctors, we don't think it's acceptable. For instance, we're in a room without air conditioning, and we're just in an interview. How about a sick person, who is in pain? Sometimes, you can't find a sterilization device, or you find it broken without alternatives.”* Medical consultant in obstetrics/gynecology, Aden

### 5.4 Transportation challenges and roadblocks

Transportation challenges due to road closures and roadblocks were additionally cited as formidable challenges. Many providers described how roadblocks erected by warring parties evoked feelings of isolation and disconnectedness, with one provider citing a feeling of being “separated from the world” due to the roadblocks. Others reported being unable to get to work without having to walk on foot for hours. While traveling to work, some participants described their encounters with armed forces at checkpoints who would deliberately obstruct them upon knowing they were health workers. Others described having to negotiate passage by displaying IDs or showing medical equipment like stethoscopes.

A hospital administrator described the challenges faced by his staff to come to work. After being identified as a health worker, one of his staff members was forced to walk on foot:

*“‘We don't want to come and risk our lives'. A doctor would tell you, ‘I have to carry stones in my purse’. Another would tell me, that they tell her to walk on foot, after checking her ID noticing she's a doctor. The rest would return administrators and so on. Some would forget their IDs. When some come to me saying they're not working anymore, I can't tell them not to come. I can't shut the hospital as it’s for the poor. This place is for the poor and the tough cases.“-* Hospital Manager, Aden

Road closures and fuel shortages also had implications for patient referrals. There were several examples of providers using their personal cars to transfer patients in need of referrals. Ambulances were either not available or rendered useless because of the chronic unavailability of fuel. In turn, this augmented health workers' feelings of isolation and helplessness. One health worker in Sana'a described the distress and uncertainty she feels when referring patients to other hospitals for services that are unavailable in her facility:

*“How can I make sure that the referred case will get to the hospital when roads are closed? I could barely find a bus to drop me here. If we meet severe cholera patients, we refer them to Al-Sabien Hospital. But we never get feedback. We do not know if the patient reaches the hospital or not. I remember once I referred a patient with severe pneumonia to Al-Sabien. I think he died”* Deputy Hospital Manager, Sana'a

## 6 Coping mechanisms

In this section, we present themes related to coping strategies emerging from health workers' accounts. These measures were employed to mitigate the impact of conflict on participants’ personal and professional lives.

### 6.1 Fatalism and religious motivation

Although providers did not report faith as an explicit coping strategy, it was evident from interviews that an important source of coping and motivation stemmed from participants’ religious beliefs. Many providers saw their service as a religious duty rewarded by God. The use of religious terminology was pervasive, and participants repeatedly invoked religious statements to make meaning out of their experiences and assert their religious duty to help others.

In affirming his duty to provide care, one participant cited a Quranic verse which states that “anyone who saves a life; it shall be as though he had saved the lives of all mankind”. Another explained, “Yes, there were no rewards or honors from anyone, but we are only working for God”. Participants also cited feeling protected by God. One participant said, “It is true that the bombing is close to us but Allah, he protected us,” and another stated, “the military police should be our protector in the first place, but Allah is our only protector”.

Additionally, a sense of fatalism pervaded provider accounts. While sometimes signaling passivity and powerlessness, more often it signified an attempt by participants to resist hopelessness and desperation. Participants recurrently resigned their fate and that of their patients to God. They acknowledged the dangerous circumstances in which they worked but felt that their fate was controlled by divine decree. This sentiment helped many cope with matters they felt they had no control over. For example, while describing shortages in medicine, one participant in Sana'a emphasized that there is only so much that he can do and the rest “he leaves for Allah's mercy”. Another provider remarked that his facility was “not fit to deliver service” and so providers had to “do what we can with what we have and rely on Allah for deliverance”. Speaking to the threats and intimidation they received from thugs and militias, the director of a hospital in Aden noted that their life was entirely “in the hands of Allah,” and so they were not afraid. Across interviews, participants repeatedly echoed the sentiment that “fate is the domain of god” and drew upon faith to help them accept their circumstances.

### 6.2 Duty to serve

The commitment of health workers to serve their country additionally emerged as a key source of motivation and a driving force to report for duty in the midst of violence and uncertainty. Many health workers asserted that their desire to serve their country and their perceived sense of duty eclipsed fears over personal safety and prompted them to show up to work despite the dangers they faced. Although some admitted that they at times stopped going to work when violence peaked, they explained that for the most part, they went back to work immediately after the violence subsided or tried to assist remotely out of obligation.

Some health workers derived a sense of purpose from feeling like they are the only option available for patients. One provider described his feeling of duty and explained that if he were to stop showing up to work, then patients would find no one else to go to because his hospital is the only functional one in the area. By focusing on patients, he was able to “overcome the problems that were disrupting services by caring for the patient only and ignoring the chaos”. Another provider explained that by “providing services to the patients, ignoring all these threats” they were able to honor their duty to serve.

### 6.3 Coping with shortages in supplies and inadequate infrastructure

On several occasions, providers demonstrated impressive resourcefulness, citing a range of creative strategies aimed at addressing the deterioration in infrastructure and shortage in supplies that they faced daily. One provider described challenges securing oxygen cylinders and masks, explaining “we do not have an oxygen mask, so we had to make one of the plastic vials. We also suffer from a severe shortage of oxygen cylinders, so we make tubular connections for more than one patient from a single oxygen cylinder.” In Taiz, a provider described the use of camels and donkeys to transfer oxygen and evade the siege.

Other coping mechanisms included staff bringing “methane gas cylinders from [their] houses and installing them in the vaccine refrigerators”, using “ice packs to keep the vaccines cold”, venturing to fix generators themselves, asking patients to leave supplies behind: “if a patient is leaving, we may ask him to leave supplies to another patient”. Others reported paying for medication themselves, or “even paying out of salaries to fix the broken equipment”. One health worker explained that health workers in his facility improvised when possible, “attempting to overcome the lack of medicines by prescribing alternative medicines or herbal treatment for patients".

## 7 Discussion

**Fig. 2:**
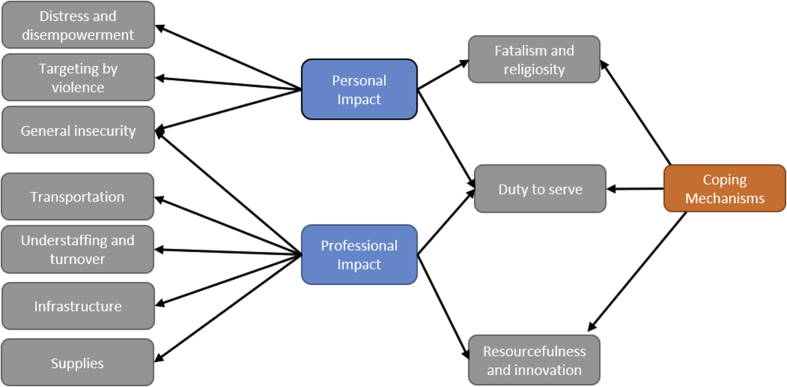
Diagrammatic illustration of the relationship between themes and subthemes emerging from the analysis.

This article contributes to a more holistic understanding of the extent and range of violence confronting health workers in conflict settings. It also sheds light on the complex challenges – both personal and professional – faced by frontline health workers, and some of the coping strategies they use to attenuate the impact of acute and chronic stressors resulting from conflict. (Fig. 2) Amid violence and insecurity, providers in Yemen bear a double burden, striving to provide care while they themselves are subjected to substantial violence.

Clearly evident from the data are the various ways in which conflict impacts and undermines health workers' wellbeing and safety. Consistent with other studies conducted in conflict settings, physical attacks, threats, and obstruction by armed factions were among the documented ways through which the conflict directly affected health workers' lives (Footer et al., 2014, 2018; Witter et al., 2017). These manifestations of violence can be understood as part of a broader pattern of ‘generalized violence’ against healthcare which Rubenstein and Bittle (2010) find are a distinguishing feature of several recent armed conflicts.

In Yemen, ‘macro-level’ drivers of violence – exemplified by a complex security situation with multiple parties to the conflict, politicization of humanitarian aid, and constraints in humanitarian access – were coupled with everyday ‘micro- and meso-level’ violence, such as sporadic attacks by armed civilians, tensions with patients, and harassment at checkpoints that impeded health workers from reaching their destination (Foghammar et al., 2016). Assaults on medical workers and facilities occurred in conjunction with changes in everyday working conditions. Economic decline reduced accessibility to food and fuel, rendering the limited goods available unaffordable for the majority of the population – including health workers. These developments in turn accentuated health workers' sense of despair and bred tensions with their patients. An increasingly armed civilian population and the proliferation of small arms further exacerbated tensions and resulted in clashes at health facilities. As highlighted in a recent Lancet editorial, the combination of political turmoil, conflict, and social grievance can result in a dangerous loss of trust in the health system (The Lancet Infectious Diseases, 2019). This lack of trust can have serious implications for healthcare demand and utilization.

In response to this climate of generalized insecurity and violence, many health workers illustrated resilience, employing a range of implicit and explicit coping strategies to mitigate the effects of the conflict both on their individual wellbeing and on the broader health system.

In responding to stressors induced by conflict, many health workers used a variety of problem-focused coping strategies to mitigate disruptions to healthcare services caused by shortages in staff, supplies, and medication (Lazarus, 1984). These included being inventive and devising improvised solutions. They also used personal assets and skills to solve problems confronted at the workplace. Similar coping strategies were highlighted in the context of the Syria crisis, whereby health workers employed innovative measures such as devising ways to make normal saline and using urine bags with anticoagulants in lieu of blood bags to cope with the dearth of supplies caused by the conflict (Fouad et al., 2017).

In addition to problem-focused coping strategies, a salient form of coping among health workers was meaning-focused (Folkman, 2008; Lazarus, 1984), whereby participants drew on internalized beliefs and value systems to sustain well-being during adversity. Health workers leveraged religious beliefs and feelings of duty to cope with the pervasive destruction around them. They repeatedly invoked “the will of God” and articulated religious sentiments imbued with fatalism to make meaning of their circumstances. While fatalism is sometimes associated with passivity and hopelessness in the public health literature (Hamdy, 2011; Nageeb et al., 2018), our findings imply that fatalism was part of a wider religious worldview that motivated perseverance and steadfastness in the face of adversity. This finding echoes insights from other studies on the role of religious coping in comparable settings (Aflakseir and Coleman, 2009; Witter et al., 2017). For example, Witter et al. (2017) found that in addition to seeking external sources of social support – a form of emotion-focused coping – health workers tended to draw on internalized value systems such as fatalism to cope with stressful and traumatic situations in the context of the Ebola outbreak, and to a lesser extent, in conflict settings. Apart from serving as coping strategies, intrinsic factors are also important sources of motivation for health workers. Indeed, a body of literature supports the importance of intrinsic motivation in stimulating worker motivation in stable settings (Franco et al., 2002; Leonard and Masatu, 2010). Our findings confirm that intrinsic motivation of health workers is particularly salient in contexts of conflict where feelings of altruism and duty appear to be amplified.

The results also highlight ways in which conflict negatively impacts health workers' self-efficacy and perceptions of their ability to perform their jobs. This is noteworthy because self-efficacy has been linked to several positive outcomes and its absence may exacerbate health workers' vulnerability to burn-out and stress. Previous studies have confirmed the link between self-efficacy of workers in humanitarian settings and their overall resilience. For example, a study of Palestinian humanitarian workers found that higher self-efficacy among workers exposed to repeated traumatic events was associated with overall higher resiliency scores (Bonnan-White and Issa, 2016). Self-efficacy has also been linked to health workers’ ability to manage work related stress and avoid exhaustion. In a Dutch study of job demands, Xanthopoulou et al. (2007) found that personal resources, including self-efficacy, mediate the relationship between workplace exhaustion and limited job resources. A literature review of workplace psychological detachment, which is the ability of workers to avoid work-related activities and thoughts during non-work times, found that the higher the level of self-efficacy a worker has, the more likely they are to have workplace detachment, which is instrumental in recovering from work related stress (Sonnentag and Fritz, 2015).

Despite some manifestations of resilience and attempts at coping with adversity among participants, the combination of micro- and macro-level drivers of violence in Yemen underpinned a general climate of stress and feelings of helplessness, inadequacy, and despair among many health workers. The finding that health workers are exposed to a broad range of chronic and acute stressors supports the notion that health personnel constitute an important affected population in conflict settings, with overlooked needs for psychological care and support. This finding resonates with other studies exploring the impact of conflict and other acute emergencies on health workers' wellbeing, and highlights the need to both effectively manage the security of health workers and to attend to their psychosocial needs. (Fouad et al., 2017; McMahon et al., 2016; Witter et al., 2017). While guidance by WHO and others directly addresses the heightened mental health needs of populations impacted by armed conflicts and natural disasters, there is an evident gap in guidelines that explicitly address the mental health needs of health workers in these same contexts (Inter-Agency Standing Committee, 2009; Ventevogel et al., 2015; WHO and UNHCR, 2015). The need to identify effective and culturally appropriate psychosocial and clinical interventions is therefore critical. Such interventions should harness providers’ current adaptive coping capacities while drawing on the evidence-base for what works to address common mental health issues that arise in humanitarian settings, such as anxiety, prolonged grief, depression, and traumatic stress disorder.

It is important to consider these findings in light of limitations. For one, the data collection took place in late 2018, and thus reflects the experiences of health workers at that point in the conflict. Since then, there have been some favorable developments which may have positively impacted health worker experiences, such as resumption of salaries in some areas in the country and improvement in the availability of supplies and medicines (Albashiri, 2019; Health Cluster Yemen, 2020; World Bank Group, 2020). Still, since 2018, attacks on the health system have increased in frequency, inflation has continued to be substantially high, and the coronavirus pandemic has further overstretched the health system (International Monetary Fund, 2020; MSF, 2020a,b). Second, the results reflect perspectives of public sector health workers only, and thus may not be reflective of the totality of health worker experiences in Yemen. However, given the volatility of the security situation in Yemen, it is reasonable to expect that these findings apply to a broad range of health workers, in both public and private sectors. Caution must also be exercised when extending these findings to other populations and settings. Furthermore, this analysis is part of a broader study on RMNCAH + N service delivery. Since the original study was not designed with the explicit aim of understanding the impact of the conflict on the lived experiences of health workers, there are inherent limitations to our data. Thirdly, our data captures the experiences of health workers who remained in service, which may be different for those who left (survivor bias). At the same time, the study has several strengths. A large number of health workers were purposively sampled and interviewed, representing those working in both IRG- and Houthi-controlled areas. Additionally, all data collection was carried out by local researchers who were well acquainted with the context. Peer debriefing and review took place to validate the findings of the study. To our knowledge, this is the first study that provides an in-depth analysis of the lived experience of Yemeni health workers serving in areas affected by the conflict.

In conclusion, our study highlights the ways in which the conflict has permeated the lives of health workers across Yemen. Health workers in humanitarian settings are exposed to a range of direct and indirect impacts of conflict and violent insecurity, underscoring the need to ameliorate the conditions in which they live and work. While they exhibit considerable resilience and coping, they have needs that remain largely unaddressed. The findings of this study serve to deepen our understanding of the subjective experiences of health workers and shine a spotlight on the need to address the mental health and psychosocial needs of health workers serving in crisis settings. The humanitarian community needs to do more to ensure that health workers are supported and protected both on the job and in the communities in which they live and serve.

## 8 Declarations

### 8.1 Ethics approval

The Johns Hopkins Bloomberg School of Public Health determined that this study was not human subjects research and therefore did not require institutional review board oversight (IRB 8665). In Yemen, the study protocol was reviewed and approved by the Research and Ethics Committee at the University of Aden School of Medicine and Health Sciences (REC-33-2018). Oral consent was obtained from all study participants before initiating data collection.

## Credit author statement

Shatha Elnakib Conceptualization, Methodology, Formal analysis, Data curation, Writing – original draft, Writing – review & editing, Sarah Elaraby Conceptualization, Methodology, Formal analysis, Data curation, Writing – original draft, Writing – review & editing, Fouad Othman Methodology, Validation, Investigation, Formal analysis, Writing – review & editing, Huda BaSaleem Methodology, Validation, Investigation, Formal analysis, Writing – review & editing, Nagiba AlShawafi Methodology, Validation, Investigation, Formal analysis, Writing – review & editing, Iman Al-Gawfi Validation, Investigation, Writing – review & editing, Formal analysis, Fouzia Shafique Writing – Review & Editing, Funding acquisition, Eman Al-Kubati Writing – Review & Editing, Supervision, Formal analysis, Nuzhat Rafique Writing – Review & Editing, Supervision, Formal analysis, Hannah Tappis Conceptualization, Methodology, Formal analysis, Data curation, Writing – original draft, Writing – review & editing, Supervision, Project administration, Funding acquisition

## Authors’ contributions

HT, SE and SE conceptualized the study. NAAA, IASAG, HBS and FO led data collection. SE and SE conducted qualitative data analysis. HT, SE, SE, FO, NAAA, HBS, IASAG, EAK, NR and FS contributed to data analysis and interpretation. SE, SE and HT drafted the initial manuscript. All authors contributed to manuscript revision and have approved the final version.

## Funding

Funding for this study was provided through a subgrant from the Centre for Global Child Health at the 10.13039/501100006126Hospital for Sick Children (SickKids) (129570), with travel and meeting costs supported directly by Aga Khan University and the Partnership for Maternal, Newborn and Child Health (PMNCH). As coordinator of the BRANCH Consortium (Bridging Research & Action in Conflict Settings for the Health of Women & Children), the SickKids Centre for Global Child Health has received funding for BRANCH research activities from the 10.13039/501100000193International Development Research Centre (IDRC) (108,416–002 & 108,640–001), the 10.13039/100007843Norwegian Agency for Development Cooperation (Norad) (QZA-16/0395), the 10.13039/100000865Bill & Melinda Gates Foundation (OPP1171560), and 10.13039/100006641UNICEF (PCA 20181204). Aga Khan University has received funding for BRANCH activities from the Family Larsson-Rosenquist Foundation. The funders had no role in study design, data collection and analysis, or preparation of the manuscript.

## Availability of data and material

Data is available from the corresponding author upon reasonable request and signature of a data sharing agreement.

## Declaration of competing interest

The authors declare they have no competing interests. The opinions expressed are those of the authors and do not necessarily reflect the views of the funding agencies.
